# Serum uric acid is associated with disease severity and may predict clinical outcome in patients of pulmonary arterial hypertension secondary to connective tissue disease in Chinese: a single-center retrospective study

**DOI:** 10.1186/s12890-020-01309-1

**Published:** 2020-10-19

**Authors:** Jingya Wang, Yuanyuan Wang, Xiaodi Li, Yingheng Huang, Xiaoxuan Sun, Qiang Wang, Miaojia Zhang

**Affiliations:** 1 Department of Rheumatology, The First Affiliated Hospital of Nanjing Medical University, Nanjing, China; 2 Xuzhou Central Hospital, Xuzhou, China

**Keywords:** CTD-PAH, Serum uric acid, Severity, Prognosis

## Abstract

**Background:**

Previous studies have shown that serum uric acid (UA) levels are correlated with the severity of idiopathic pulmonary arterial hypertension (IPAH) and are predictors of disease prognosis. Still, few studies have explored the value of serum UA in pulmonary arterial hypertension secondary to connective tissue disease (CTD-PAH). This retrospective study aimed to investigate the clinical value of serum UA levels in patients with CTD-PAH.

**Methods:**

Fifty CTD-PAH patients were enrolled in our study, from which baseline UA levels, respective variations, and additional clinical data were collected. The potential association between baseline UA level and severity of CTD-PAH was investigated. Furthermore, the relationship between baseline UA and survival rate of CTD-PAH patients, as well as between UA variations and survival rate of pulmonary hypertension secondary to connective tissue disease (CTD-PH) patients was discussed.

**Results:**

Baseline serum UA levels were positively correlated with pulmonary vascular resistance (PVR). During the follow-up period, 3 CTD-PAH and 12 CTD-PH patients died. Kaplan-Meier survival curves showed lower survival rate in patients with hyperuricemia than in patients with normouricemia, in both groups (CTD-PAH group *p* = 0.041, CTD-PH group *p* = 0.013). Concerning serum UA variations, patients with persistent hyperuricemia showed the lowest survival rate when compared with patients with steady normouricemia (*p* = 0.01) or patients with decresing serum UA levels, i.e. undergoing from a status of hyperuricemia to a status of normouricemia (*p* = 0.023).

**Conclusion:**

Baseline serum UA levels might predict severity of CTD-PAH. Together with baseline values, changes of uric acid level may predict the clinical prognosis of the disease.

## Introduction

Pulmonary arterial hypertension (PAH) is a life-threatening and refractory disease, associated with progressive pulmonary vascular remodeling and increased pulmonary vascular resistance, ultimately leading to right ventricular failure and death. Connective tissue disease (CTD) is the primary cause of PAH [1]. In turn, PAH is the third cause of death among CTD patients, highlighting the impoatance of a proper evaluation of disease severity and prognosis. Serum uric acid (UA), the end product of purine metabolism, has been purposed as a prognostic marker of pulmonary arterial pressure [2–4], which is related with PAH aggressiveness, [5–13], and has been reported to predict the clinical outcome of the disease [6, 8, 13–19]. However, serum UA can be generated in varying contexts, such as cardiac overproduction, renal impairment, diet, or use of diuretics in right-sided heart failure. Therefore, the use of UA as an indicator of PAH severity and prognosis remains controversial. The association between UA and CTD-PAH has been previously described, and up to date, the evidence suggests that: 1) UA levels above 357 μmol/L are associated with PAH in systemic lupus erythematosus (SLE) [20]; 2) Serum UA levels are correlated with disease severity in PAH secondary to systematic sclerosis (SSc-PAH) [21]; 3) Serum baseline UA levels may predict clinical outcome in patients with CTD-PH [18]. In this context, the authors concluded that UA levels were correlated to pro-BNP and SPAP. In contrast, the relationship between serum UA levels and hemodynamic parameters in CTD-PAH patients was rarely discussed. Moreover, the numerical criteria for defining pulmonary hypertension (PH) are inconsistent with each other. Finally, it is still to uncover the association of UA concentration variations and the prognosis of CTD-PH patients. Therefore, we performed a retrospective study, to disclose the correlation between baseline UA levels and CTD-PAH severity and prognosis. We further studied the clinical relevance of UA variations to the outcome of CTD-PH patients.

## Methods

### Design

CTD-PAH patients were evaluated and treated in the rheumatology department in the first affiliated hospital of Nanjing Medical University during the period of 2009–2018. This study was approved by the Medical Ethics Committee of the First Affiliated Hospital of Nanjing Medical University (number 2018-SR-333).

### Patients

**Fig. 1:**
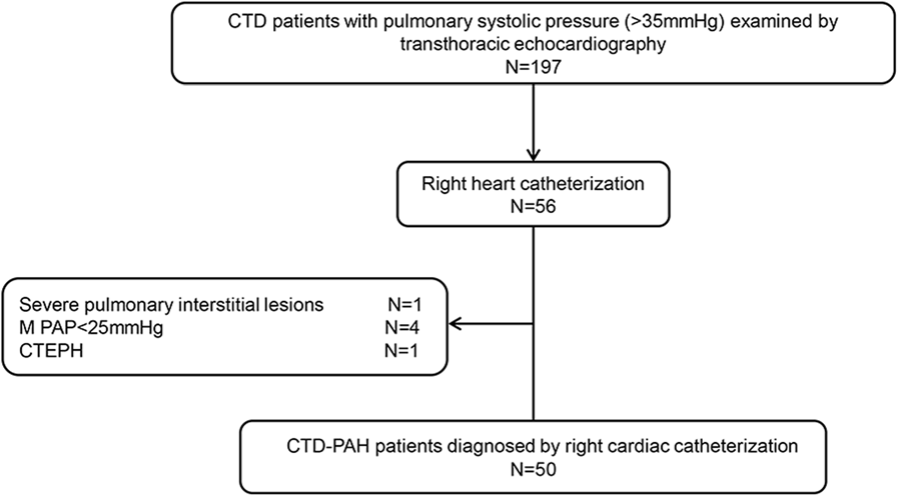
Inclusion flow chart of CTD-PAH patients diagnosed by RHC

In this study, 197 patients were screened, and according to the inclusion/exclusion criteria, and 50 patients were enrolled. The screening process was as follows (Fig. 1). Inclusion criteria: a. compliance with the diagnostic criteria for connective tissue diseases. CTD were diagnosed as follows: SLE was diagnosed according to 1997 American Rheumatism Association (ACR) criteria, primary Sjogren’s syndrome (pSS) was defined according to 2002 international classification criteria, systematic sclerosis (SSc) was defined according to 1980 ACR criteria, mixed connective tissue disease (MCTD) was defined by Sharp criteria. b. diagnosis of pulmonary hypertension by Right-heart catheterization (RHC): mean pulmonary arterial pressure (m PAP) ≥ 25 mmHg at rest, pulmonary arterial wedge pressure (PAWP) ≤ 15 mmHg and pulmonary vascular resistance (PVR) ≥ 3 Wood Units. Exclusion criteria: a. patients with significant interstitial lung disease (ILD) or chronic obstructive pulmonary disease based on the results of chest high-resolution computed tomography (HRCT), b. evidence of pulmonary venous hypertension (pulmonary capillary wedge pressure > 15 mmHg), c. chronic thromboembolic disease, based on the results of computed tomography angiography, d. patients with a history of IPAH, drug or toxin exposure, human immunodeficiency virus (HIV) infection, portal hypertension or any other diseases known to be associated with pulmonary hypertension (PH), e. patients with gout, f. patients with renal insufficiency, g. systemic vasculitis was excluded.

Hyperuricemia was defined as serum UA levels of women ≥357 μ mol/L and men ≥420 μ mol/L.

### Data collection and outcome assessment

We used a uniform evaluation chart to collect patient information, which included demographic data, clinical manifestations, laboratory results, and treatment regimens. Demographic data included gender, age, height, weight, PAH symptoms at the diagnosis of CTD-PAH with right cardiac catheter. Recruitment date was defined as the RHC date for CTD-PAH patients. As for treatment regimens, steroid and immuno-suppressors were documented for the dosage and administration route. Use of diuretics and PAH-targeted medicine were also recorded and analyzed. Blood samples for baseline measurements were obtined from a peripheral vein within 1 week of the first diagnostic catheterization from hemodynamically stable patients not receiving specific PAH drugs. UA, creatinine, fasting glucose, N-terminal pro-B-type natriuretic peptide (NT-pro BNP) and autoantibodies were measured in the central laboratory of the first affiliated hospital of Nanjing Medical University. Transthoracic echocardiography was examined in all suspicious PAH patients. RHC was performed in 50 patients during hospitalization. The baseline hemodynamic parameters were measured in all patients.

To study disease progression, we performed outpatient follow-up and telephone follow-up. The first was performed every 3–6 months, to collect the clinical symptoms, the results of cardiac ultrasound and the levels of uric acid of the patients. Telephone follow-up was used to monitor clinical outcome. The primary endpoint was all-cause mortality. The survival rate of CTD-PAH patients was estimated from the date of first catheterization until 2 years, or the date of death.

### Statistical analysis

**Fig. 2:**
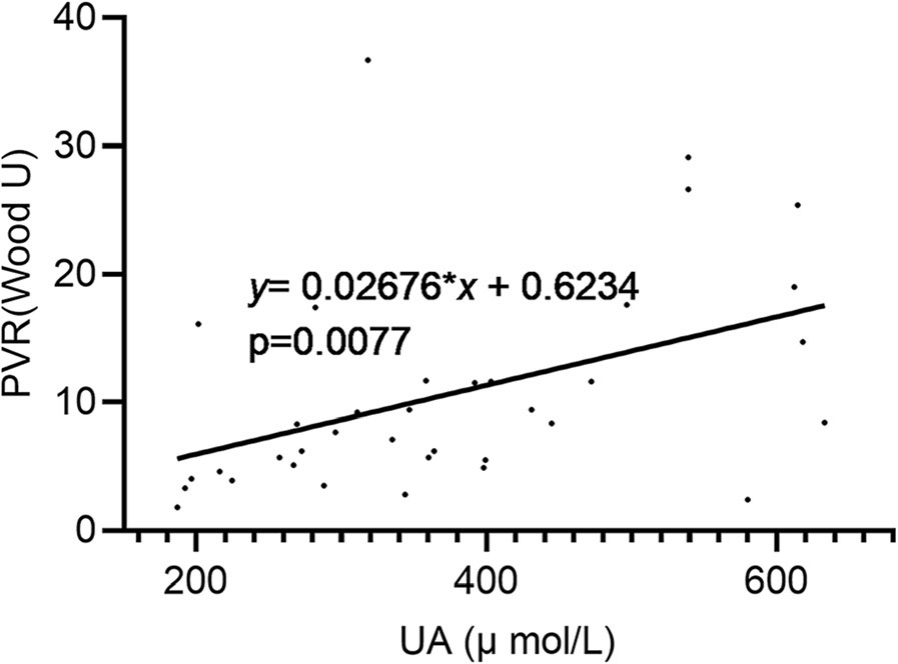
Linear correlation analysis between UA and PVR

Numerical values were expressed as mean ± standard error. Comparisons between two groups were calculated by Student’s unpaired *t* test or Fisher’s exact test. Pearson correlation analysis was used to evaluate the correlation between the indicators. Univariate correlation analysis and linear regression were used to analyze the related factors of elevated UA level. Survival curves were derived using the Kaplan-Meier method and were compared using log-rank test. *P* value < 0.05 was considered statistically significant. Statistical analyses were performed using SPSS.V20 or GraphPad Prism 8.0, Fig. 2 was made with GraphPad Prism 8.0, and all the survival curves were drawn in the Sanger box.

## Results

Fifty patients with CTD-PAH (47 women, average age 38.5 ± 0.3 years, 3 men, average age 49.3 ± 13.7 years) were included in this study.

### Baseline characteristics of the normouricemia and the hyperuricemia groups (Table 1)

The 50 patients were separated into two groups based on serum UA levels: normouricemia group (UA: women < 357 μmol/L, men < 420 μmol/L) and hyperuricemia group (UA: women ≥357 μmol/L, men ≥420 μmol/L). Compared with normouricemia group, superior vena cava pressure and PVR levels were significantly higher in the hyperuricemia group. Cardiac output (CO), cardiac index (CI) and mixed venous oxygen saturation (SVO_2_) were significantly smaller in the hyperuricemia group. However, there were no significant differences between the two groups regarding demographic characteristics, clinical symptoms, World Health Organization Cardiac Function Classification (WHO-FC), parameters of ECHO, diuretics.

**Table 1:** Baseline Characteristic of normouricemia and hyperuricemia groups

	Hyperuricemia group(*n* = 23)	Normouricemia group (*n* = 27)	p
Gender (women/ men)	21/2	26/1	
Age, Y	40.17 ± 3.11	38.22 ± 2.36	
SLE	7 (14%)	13 (26%)	
SS	8 (16%)	6 (12%)	
CTD	4 (8%)	0	
SSc	3 (6%)	2 (4%)	
SLE/SS	1 (2%)	1 (2%)	
MCTD	0	5 (10%)	
Body Mass Index, kg/m^2	22.0 ± 0.1	22.28 ± 0.71	ns
WHO functional class, III-IV/I-II	15/8	17/10	ns
6MWD, m	390.8 ± 30.85	404.1 ± 27.93	ns
UA, μmol/L	467.0 ± 19.67	277.7 ± 10.02	ns
Creatinine, μmol/L	63.24 ± 3.02	63.14 ± 5.33	ns
NT-pro BNP, pg/ml	2368 ± 662.80	1270 ± 465.80	ns
Treatment with diuretics, yes/no	5/18	6/21	ns
echocardiography			
PASP, mmHg	83.13 ± 4.93	74.19 ± 3.40	ns
TAPSE, cm	1.53 ± 0.11	2.15 ± 0.36	ns
right heart catheterization			
Rap, mmHg	8.13 ± 1.22	6.00 ± 0.91	ns
mPAP, mmHg	45.43 ± 2.41	45.70 ± 2.09	ns
PAWP, mmHg	8.91 ± 0.76	8.58 ± 0.77	ns
CO, L/min	3.75 ± 0.37	5.19 ± 0.51	0.029
CI, L/min/m^2^	2.31 ± 0.24	3.15 ± 0.28	0.028
PVR, Wood U	12.77 ± 1.85	8.50 ± 1.93	0.0244
SVO2, %	57.81 ± 2.51	65.13 ± 1.97	0.029

Numerical values were expressed as mean ± standard error. Comparisons between two groups were calculated by Student’s unpaired *t* test or Fisher’s exact test

*SLE* systemic lupus erythematosus, *SS* Sjogren’s syndrome, *CTD* connective tissue disease, *SSc* systematic sclerosis, *SLE/SS* systemic lupus erythematosus /Sjogren’s syndrome, *MCTD* mixed CTD (MCTD) was defined by Sharp criteria, *WHO functional class* World Health Organization Cardiac Function Classification, *6MWD* 6-min walking distance, *NT-pro BNP* N-terminal pro-B-type natriuretic peptide, *PASP* pulmonary artery systolic pressure, *TAPSE* systolic displacement of tricuspid annulus, *RAP* right atrial pressure, *mPAP* mean pulmonary arterial pressure, *PAWP* pulmonary artery wedge pressure, *CO* cardiac output, *CI* cardiac index, *PVR* pulmonary vascular resistance, *SVO2* mixed venous oxygen saturation

### Baseline serum UA levels and severity in CTD-PAH

In univariate correlation analysis of variables associated with serum UA levels in CTD-PAH, baseline UA levels were positively correlated with pulmonary artery systolic pressure (PASP), right atrial pressure (RAP), PVR, creatinine and NT-pro BNP, and negatively with CI and CO (Table 2). In linear regression, PVR correlated to baseline serum UA levels (adjusted R^2^ = 0.163, (Table 3). Linear correlation analysis showed that PVR increased by 0.02676 Wood U with the increase of 1 μmol/L of UA levels (Fig. 2).

**Table 2:** Analysis of variables associated with serum uric acid levels in patients of CTD-PAH

	R squared	Pearson r	95% confidence interval	*P* value
Age	0.004175		−2.017 to 3.174	0.6557
BMI	0.00257		−0.3424 to 0.2499	0.7438
FBS	0.001525		−0.2842 to 0.3543	0.8159
creatinine	0.3106		0.2891 to 0.7444	0.0003
WHO functional class, I/II/III/IV	0.04782		−0.06354 to 0.4685	0.1271
6MWD	0.09445		−1.204 to 0.3133	0.2302
NT-pro BNP	0.1291	0.3593	0.05378 to 0.6034	0.0228
PASP	0.08235	0.287	0.05005 to 3.258	0.0433
Rap	0.09764	0.3125	0.02772 to 0.5503	0.0325
PAWP	0.0272		−0.1318 to 0.4345	0.2734
CO	0.1418	−0.3766	−0.6035 to − 0.09333	0.0108
CI	0.1549	−0.3936	−0.6160 to − 0.1130	0.0075
PVR	0.1908	0.4369	0.1264 to 0.6694	0.0077
SVO2%	0.1073		−0.6069 to 0.02402	0.0673

Pearson correlation analysis was used to evaluate the correlation between the indicators

*BMI* body mass index, *FBS* fasting blood sugar

**Table 3:** Linear regression analysis of variables associated with PVR in patients of CTD-PAH, the independent variable is UA

Independent variable	Non-standardization coefficient		t	Sig.
	B	Standard error		
UA	0.027	0.01	2.793	0.009

### Baseline uric acid levels and prognosis in patients with CTD-PAH

**Fig. 3:**
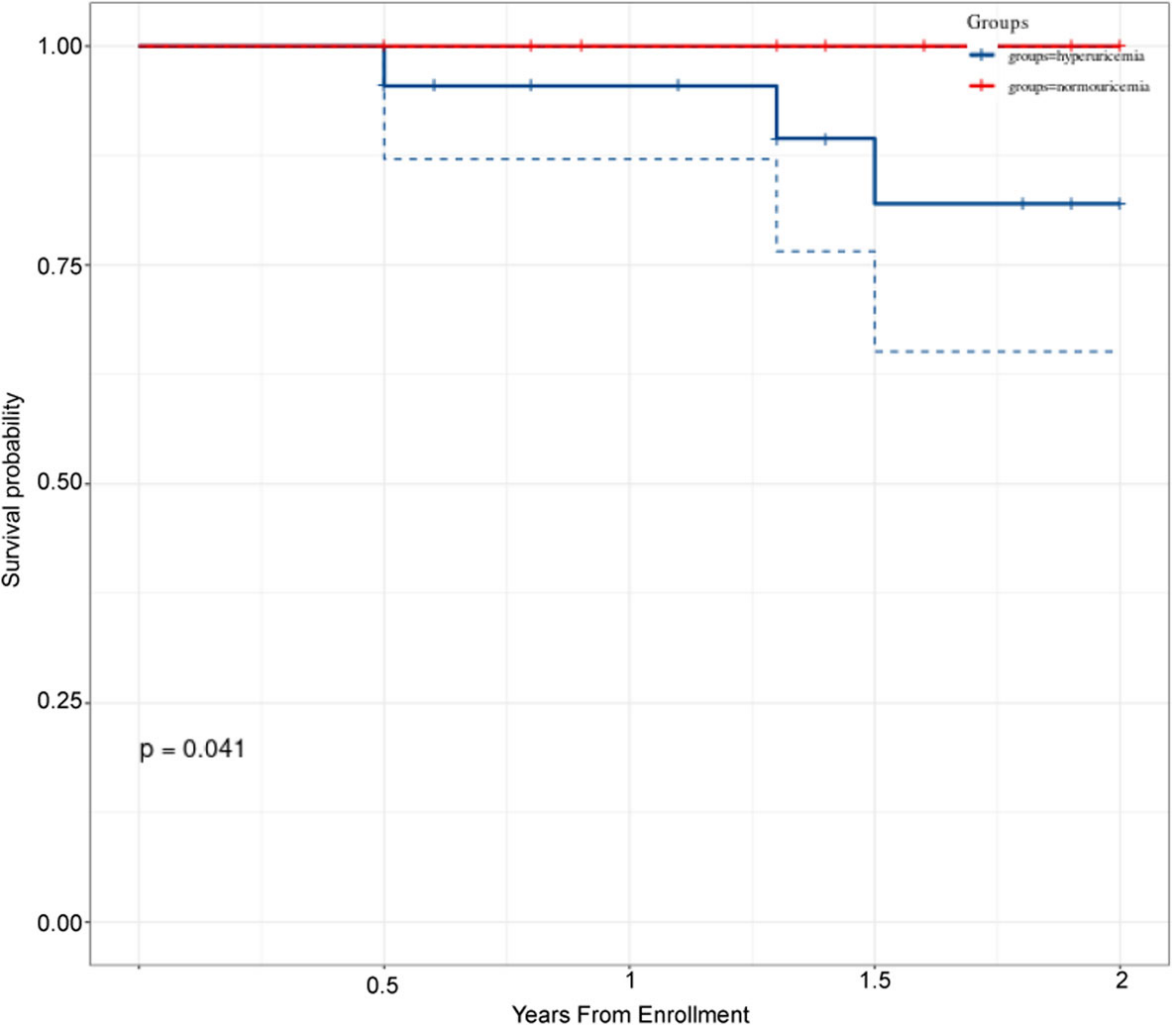
Kaplan-Meier survival curves of normouricemia and hyperuricemia groups in CTD-PAH patients (*p* = 0.041)

During the follow up period (2 years) 3 patients died and 2 patients were lost to follow-up. There were no patients receiving lung or heart-lung transplant. The overall follow-up rate was 96%. Kaplan-Meier survival curves, in accordance with the two groups of the baseline serum UA levels, revela that hyperuricemic patients have lower survival rate than normouricemic patients (*p* = 0.041, Fig. 3).

### Variation of UA level and prognosis of patients with CTD-PH

**Fig. 4:**
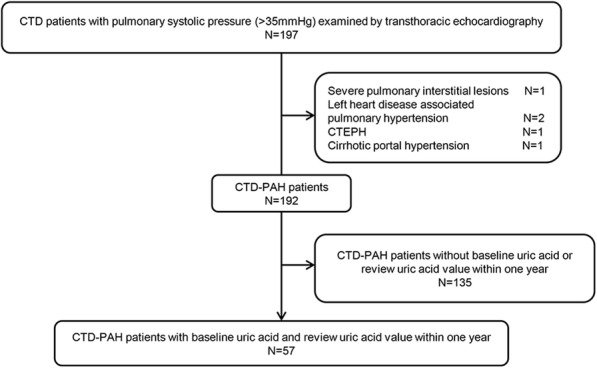
Inclusion flow chart of CTD-PH patients with variation of UA

We studied the relationship between UA level variations and the prognosis of patients with CTD-PH. Given the small the number of CTD-PAH patients with UA variations, we enrolled CTD-PH diagnosed using ultrasound. Fifty-seven patients with CTD-PH diagnosed by ECHO were enrolled in this part (Fig. 4).

**Fig. 5:**
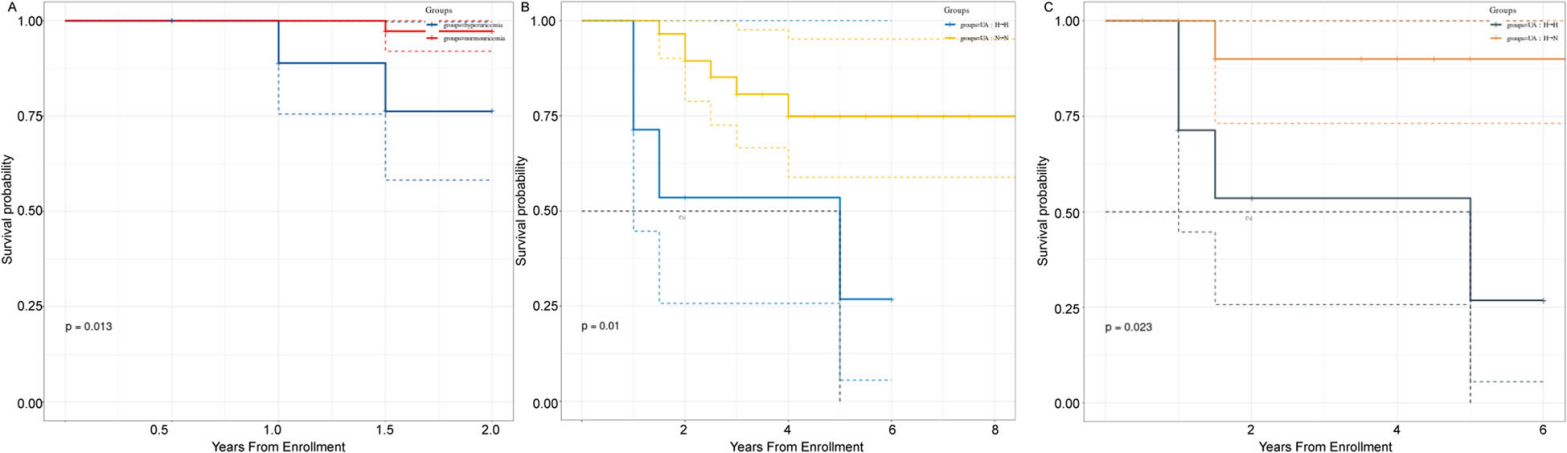
**a** Kaplan–Meier survival curves for normouricemia and hyperuricemia groups according to the respective baseline UA levels in CTD-PH patients (*p* = 0.013); **b** Kaplan–Meier survival curves of patients with steady hyperuricemia (UA: H → H) and patients with steady normouricemia (UA: N → N); *p* = 0.01; **c** Kaplan–Meier survival curves of patients with steady hyperuricemia (UA: H → H) and patients with decreasing uricemia during the follow-up period (UA: H → N), *p* = 0.023

We first whether ther is some association between baseline UA and the prognosis of CTD-PH patients. The 57 patients were separated into two groups based on baseline serum UA levels: normouricemia group (UA: women< 357 μmol/L, men < 420 μmol/L) and hyperuricemia group (UA: women ≥357 μmol/L, men≥420 μmol/L). During the follow up period (2 years) 12 patients died and 2 patients were lost to follow-up. There were no patients receiving a lung or heart-lung transplant. The overall follow-up rate was 96.49%. Kaplan-Meier survival curves, in accordance with the two groups of the baseline serum UA levels, revealed that hyperuricemic patients has lower survival rates than normouricemic patients (*p* = 0.013, Fig. 5a).

We further studied the relationship between UA level variation and the clinical outcome of CTD-PH patients, by performing a UA reexamination from 6 months to 1 year after the diagnosis of PH by echocardiography. Patients were then divided into 4 groups, according to UA levels at baseline and respective variations as follows: steady normouricemia (normouricemia → normouricemia; UA: N → N), increasing uricemia (normouricemia → hyperuricemia; UA: N → H), steady hyperuricemia (hyperuricemia →hyperuricemia; UA: H → H) and decreasing uricemia (hyperuricemia → normouricemia; UA: H → N), respectively. Details on UA variations and the follow up of participants are described in the Table 4. The survival rates among the four groups showed significant differences (Supplementary Figure 1A, *p* = 0.022). Overall, steady heperuricemic patients showed the lowest survival rate, when compared to patients with steady normouricemia (*p* = 0.01) and patients with decreasing uricemia (UA: H → N; *p* = 0.023; Fig. 5b). In contrast there were no significant differences between the survival rate of UA: N → N and the UA: N → H groups (Supplementary Figure 1B; *p* = 0.83).

**Table 4:** Details on UA variations in CTD-PH patients

Group	UA at baseline	UA at reexamination	number at enrollment	censor	death
	(mean ± SD) (μmol/L)	(mean ± SD) (μmol/L)			
UA: H → H	509.6625 ± 124.631	519.025 ± 133.6553	8	1	4
UA: H → N	477.9083 ± 140.6724	300.7083 ± 46.3318	12	0	1
UA: N → N	260.27 ± 54.92758	256.7433 ± 71.03973	30	1	6
UA: N → H	288.4571 ± 44.99062	412.3571 ± 58.32638	7	0	1

UA: H → H = steady hyperuricemia (hyperuricemia →hyperuricemia;), UA: H → N = decreasing uricemia (hyperuricemia → normouricemia;), UA: N → N = steady normouricemia (normouricemia → normouricemia;), UA: N → H = increasing uricemia (normouricemia → hyperuricemia;)

## Discussion

In the present study, we demonstrated that (1) hyperuricemia was detected in 46% of CTD-PAH patients, (2) baseline serum UA levels are positively correlated with pulmonary vascular resistance, (3) baseline UA levels might have a prognostic value in the context of CTD-PAH and CTD-PH, at least for a period of 2 years, (4) UA level variations within 1 year might predict the long-term clinical outcome of CTD-PH patients.

Our retrospective study showed that baseline serum UA levels were positively correlated with pulmonary vascular resistance, and that pulmonary vascular resistance increased by 0.02676 Wood U with the increase of 1 μmol/L of UA levels. Pulmonary vascular resistance measured with right cardiac catheterization is a critical parameter to account for during the assessment of PAH severity [22, 23]. Endothelial dysfunction of the pulmonary artery and proliferation of pulmonary artery smooth muscle cells are important promoters of pulmonary vascular resistance. On the one hand, uric acid may cause endothelial cell dysfunction, as its biosynthetic pathway generates reactive oxygen species (ROS) as a byproduct. In hyperuricemia, the excessive production of UA is accompanied by excessive generates reactive oxygen species production, thus as UA is absorbed by endothelial cells, oxidative damage is increased. The presence of generates reactive oxygen species in the lungs might induce deterioration of endothelial function, reduced nitric oxide (NO) bioavailability [24] and increased pulmonary vascular resistance. On the other hand, in hyperuricemia conditions, UA enters vascular smooth muscle cells through an anion transporter [25] and stimulates proliferation by activating mitogen activated protein kinases (MAPK) [26], cyclooxygenase-2 (COX-2) [27], the vascular renin-angiotensin system [28] and the platelet-derived growth factor (PDGF) and PDGF receptors [29]. An interesting question is whether UA may lead to proliferation of pulmonary artery smooth muscle cells and pulmonary vascular remodeling. In conclusion, UA may increase oxidative stress, weaken endothelial cell function and promote smooth muscle cell proliferation. Moreover, we purpose that serum UA may be used to assess the severity of CTD-PAH as well as IPAH.

In our study we observed that, compared with normouricemia group, the disease severity in conditions of hyperuricemia was increased, after 2 years of follow-up. We also observed that patients with baseline hyperuricemia had lower survival rate than those with baseline normouricemia, suggesting that baseline UA levels might predict the clinical outcome of CTD-PAH and CTD-PH patients. Our findings are consistent with previous studies showing the prognostic use of serum UA in IPAH. The levels of accurate biomarkers should fluctuate according to the course of the disease. The levels of serum UA are reported to decrease after PAH improvement [30]. Previous studies have also suggested that changes in serum uric acid levels might influence the outcome of pediatric PAH [31]. In this study, we observed that UA variation levels influenced the course of the disease, with steady hyperuricemic patients showing an overall lower survival rate that patients with steady normouricemia or patients with decreasing UA levels during the follow-up period. We also observed that the UA variations within 1 year may predict long-term survival rate of CTD-PH patients more accurately than baseline UA levels. Although we did not find significant differences in the survival rates between patients with steady normouricemia (UA: N → N) and patients with increasing uricemia (UA: N → H). During the follow-up period, our results show that after 5 years, the survival rate of the later patientsis significantly lower than the first (*p* = 0.025, Supplementary Figure 1C and D). We hypothesize that our results would be enforced by including a higher number of patients in the analysis.

The results concerning the UA variations within 1 year and the survival rate of CTD-PH, displayed in Supplementary Figure 1A, show that steady normouricemic patients have lower overall survival than patients in H → N group, which is inconsistent with the results obtained so far. We hypothesize that such discrepancies might result from the fact that we have all-cause mortality as the endpoint, rather than PH specific mortality in our study. So there are other factors, such as co-infection, may also affect the survival rate of patients. Because this was a retrospective study, clinical deterioration events could not be accurately tracked, so we chose all-cause death. In future research, we will set the endpoint events as PAH or PH clinical deterioration events. Maybe, the result will be different. Indeed, this is a flaw in our research.

A previous study conducted by Simpson, and colleagues demonstrated an association between uric acid levels and disease risk, severity, and survival in systemic sclerosis-related pulmonary arterial hypertension. However, the major underlying Connective tissue diseases associated with PAH in Chinese patients appear to be systemic lupus erythematosus, systematic sclerosis, and primary Sjogren’s syndrome [32]. Accordingly, in this study systemic lupus erythematosus, systematic sclerosis, primary Sjogren’s syndrome, mixed connective tissue disease, and Connective tissue diseases are included.

Hyperuricemia might result from insulin resistance, renal dysfunction, gout, diuretic use and obesity, none of which is directly related to PAH. In this context, the value of uric acid in patients may be reduced, therefore we excluded gout and renal insufficiency at the time of inclusion. Moreover, we did not find significant differences in the level of uric acid between the patients using diuretics (*N* = 11, average uric acid level 335 μmol/L) and those who did not use diuretics (*N* = 39, uric acid level 343.8 μmol/L) when enrolled in our study (*p* = 0.5922), and there is no significant differences in BMI between patients with baseline hyperuricemia and normouricemia.

Despite our attempts to control the maximum number of variables, we consider that our report has several limitations. First, due to the small number of patients with CTD, we could not separate patients by gender. Second, because CTD-PAH is more common in young people, we could only include a small number of patients over 65 years. Thus, our study is not fully representative of the whole population. Third, because only a few patients with UA re-examination value were diagnosed by right cardiac catheterization, we included CTD-PH pateients for ultrasonographic diagnosis. Finally, we did not perform routine insulin measuements, given that only two patients included had diabetes mellitus. Moreover we identified normal levels of fasting blood glucose in all participants.

In summary, UA levels might reflect disease severity and patient prognosis. Therefore, we purpose UA levels can be used to evaluate the severity, treatment efficiency and prognosis of patients with clinically confirmed CTD-PAH.

## Supplementary information

**Additional file 1: Supplementary Figure 1.** A Kaplan–Meier survival curves of the four patient groups, according to UA level variation; *p* = 0.022; B: Kaplan–Meier survival curves of patients with increasing uricemia during the follow up period (UA: N → H) and patients with steady normouricemia (UA: N → N), *p* = 0.83; C Kaplan–Meier 5-year survival curves of patients with increasing uricemia during the follow-up period (UA: N → H) and patients with steady normouricemia (UA: N → N), *p* = 0.26; D: Kaplan–Meier 5–8 year survival curves of patients with increasing uricemia during the follow-up period (UA: N → H) and patients with steady uricemia (UA: N → N), *p* = 0.025

## Data Availability

The datasets used and analysed during the current study are available from the corresponding author on reasonable request.

## Abbreviations

UA: Uric acid
IPAH: Idiopathic pulmonary arterial hypertension
CTD-PAH: Pulmonary arterial hypertension secondary to connective tissue disease
CTD-PH: Pulmonary hypertension secondary to connective tissue disease
ACR: American Rheumatism Association
HIV: Human immunodeficiency virus
PH: Pulmonary hypertension
PVR: Pulmonary vascular resistance
PAH: Pulmonary arterial hypertension
CTD: Connective tissue disease
SLE: Systemic lupus erythematosus
SSc-PAH: PAH secondary to systematic sclerosis
ECHO: Echocardiogram
RHC: Right cardiac catheterization
pSS: Primary Sjogren’s syndrome
SSc: Systematic sclerosis
MCTD: Mixed connective tissue disease
mPAP: Mean pulmonary arterial pressure
PAWP: Pulmonary arterial wedge pressure
ILD: Interstitial lung disease
HRCT: High-resolution computed tomography
NT-proBNP: N-terminal pro-B-type natriuretic peptide
CO: Cardiac output
CI: Cardiac index
SVO_2_: Mixed venous oxygen saturation
WHO-FC: World Health Organization Cardiac Function Classification
PASP: Pulmonary artery systolic pressure
RAP: Right atrial pressure
ROS: Generates reactive oxygen species
NO: Nitric oxide
MAPK: Mitogen activated protein kinases
COX-2: Cyclooxygenase-2
PDGF: Platelet-derived growth factor
