# Supplementary figures and images for: Serum uric acid is associated with disease severity and may predict clinical outcome in patients of pulmonary arterial hypertension secondary to connective tissue disease in Chinese: a single-center retrospective study
Source: BMC Pulm Med. 2020 Oct 19;20:272. doi: 10.1186/s12890-020-01309-1 (PMC7574226; doi:10.1186/s12890-020-01309-1)

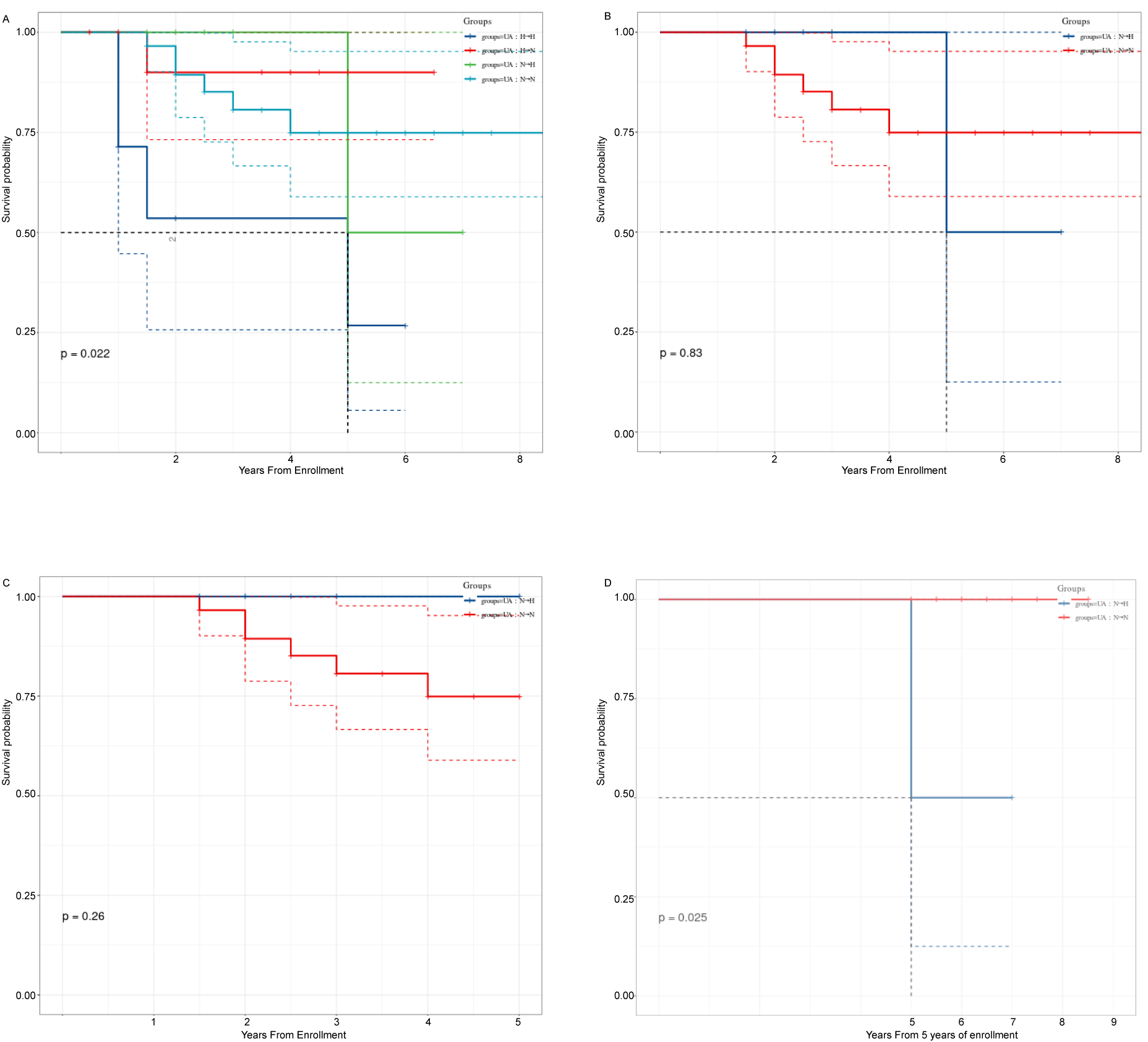

Supplement: Supplementary file 1 — Additional file 1: Supplementary Figure 1. A Kaplan–Meier survival curves of the four patient groups, according to UA level variation; p = 0.022; B: Kaplan–Meier survival curves of patients with increasing uricemia during the follow up period (UA: N → H) and patients with steady normouricemia (UA: N → N), p = 0.83; C Kaplan–Meier 5-year survival curves of patients with increasing uricemia during the follow-up period (UA: N → H) and patients with steady normouricemia (UA: N → N), p = 0.26; D: Kaplan–Meier 5–8 year survival curves of patients with increasing uricemia during the follow-up period (UA: N → H) and patients with steady uricemia (UA: N → N), p = 0.025 [file 12890_2020_1309_MOESM1_ESM.tif]
